# Participation in Community Preparedness Programs in Human Services Organizations and Faith-Based Organizations — New York City, 2018

**DOI:** 10.15585/mmwr.mm6835a2

**Published:** 2019-09-06

**Authors:** Luis Rivera, Melissa Pagaoa, Beth Maldin Morgenthau, Christopher Paquet, Noelle Angelique M. Molinari, Tanya Telfair LeBlanc

**Affiliations:** ^1^New York City Department of Health and Mental Hygiene; ^2^Division of State and Local Readiness, Center for Preparedness and Response, CDC.

Community-based organizations have a long history of engagement with public health issues; these relationships can contribute to disaster preparedness (*1*,*2*). Preparedness training improves response capacity and strengthens overall resilience (*1*). Recognizing the importance of community-based organizations in community preparedness, the Office of Emergency Preparedness and Response in New York City’s (NYC’s) Department of Health and Mental Hygiene (DOHMH) launched a community preparedness program in 2016 (*3*), which engaged two community sectors (human services and faith-based). To strengthen community preparedness for public health emergencies in human services organizations and faith-based organizations, the community preparedness program conducted eight in-person preparedness trainings. Each training focused on preparedness topics, including developing plans for 1) continuity of operations, 2) emergency management, 3) volunteer management, 4) emergency communications, 5) emergency notification systems, 6) communication with persons at risk, 7) assessing emergency resources, and 8) establishing dedicated emergency funds (*2*,*3*). To evaluate training effectiveness, data obtained through online surveys administered during June–September 2018 were analyzed using multivariate logistic regression. Previously described preparedness indicators among trained human services organizations and faith-based organizations were compared with those of organizations that were not trained (*3*). Participation in the community preparedness program training was associated with increased odds of meeting preparedness indicators. NYC’s community preparedness program can serve as a model for other health departments seeking to build community preparedness through partnership with community-based organizations.

NYC DOHMH’s community preparedness program is based on recommendations from the Federal Emergency Management Agency and CDC to engage with community partners to prepare for disasters (*1*,*2*,*4*). In 2016, NYC implemented a sector-based community preparedness program beginning with the human services and faith-based sectors. The Human Services Council and the New York Disaster Interfaith Services were selected through a competitive process to serve as lead organizations for the human services and faith-based sectors, respectively. The principal role of the human services sector is to provide social services to communities, whereas that of the faith-based sector is to provide spiritual guidance. The sector lead organizations build and strengthen partnerships within their constituents through emergency planning with community organizations to provide connections with the public health preparedness and recovery structure (*1*–*4*). The community preparedness program, in tandem with the sector lead organizations, works to expand community relationships within sectors; foster emergency planning; offer trainings; build communication capacity; and provide linkages with the local preparedness infrastructure.

DOHMH and the sector lead organizations invited 595 human services organizations and faith-based organizations within their memberships via e-mail to attend eight in-person half- to full-day preparedness trainings during April 2017–May 2018. Of these, 444 organizations attended at least one training. The trainings covered approaches for strengthening organization preparedness across key domains, including community resilience, incident management, information management, and surge management (*4*). Trainings covered continuity of operations development, communications, emergency planning, NYC City Incident Management Systems, active shooter guidance, and targeted grassroots-level preparedness (*5*). Training participants were also briefed on the NYC government’s plan to address citywide emergencies and multiple large-scale incidents, highlighting the roles and responsibilities of human services organizations and faith-based organizations as community partners during emergencies. This report presents evaluation data collected following implementation of training content.

Data obtained through online surveys sent to 850 human service organizations and 1,000 faith-based organizations during June–September 2018 (after the trainings) compared preparedness indicators among human services organizations and faith-based organizations that participated in any of the trainings with those that had not participated in any trainings. E-mail reminders were sent to targeted responders every 2 weeks to encourage participation. Multivariate logistic regression was used to estimate the odds of meeting preparedness indicators among trained human services organization and faith-based organizations compared with those that were not trained, controlling for multiple organization-specific characteristics. P-values <0.05 were considered statistically significant. All analyses were conducted using SAS (version 9.4; SAS Institute).

Overall, 115 (13.5%) human service organizations and 185 (18.5%) faith-based organizations completed the survey. Of the 115 human services organizations responding to the survey, 61 (53%) participated in at least one community preparedness program training (range = 1–8). After controlling for agency/governance type, number of staff members, number of volunteers, client volume, operating budget, borough, and religious affiliation, if any, organizations that participated in at least one community preparedness program training had significantly increased odds of having plans for continuity of operations (adjusted odds ratio [AOR] = 45.7, p<0.001), emergency management (AOR = 12.8, p<0.001), volunteer management (AOR = 6.3, p = 0.007), and emergency communications (AOR = 17.3, p<0.001) than did those that did not participate in any training (Table 1). Community preparedness program training also significantly increased the odds of having emergency notification systems (AOR = 8.7, p<0.001), inventoried emergency resources (AOR = 9.8, p<0.001), and the ability to communicate with clients at risk (AOR = 15.6, p<0.001) before, during, and after an emergency.

**TABLE 1:** Participation in community preparedness program training among human services organizations (N = 115) — New York City, 2018

Preparedness component in place	No. (%) responding “yes”		Adjusted odds ratio* (95% CI)	P-value
	Participated in any training (n = 61)	Did not participate in any training (n = 54)		
Continuity of operations plan	48 (79)	13 (24)	45.7 (10.9–191.6)	<0.001
Emergency management plan	51 (84)	22 (41)	12.8 (3.4–48.0)	<0.001
Plan for using volunteers	21 (34)	4 (7)	6.3 (1.7–24.2)	0.007
Emergency communications plan	50 (82)	18 (33)	17.3 (5.2–57.6)	<0.001
Emergency notifications system	40 (66)	18 (33)	8.7 (3.1–24.8)	<0.001
At-risk population communication	49 (80)	20 (37)	15.6 (5.1–47.6)	<0.001
Inventory of emergency resources^†^	42 (69)	12 (22)	9.8 (3.7–26.0)	<0.001
Dedicated emergency funds^§^	14 (23)	6 (11)	3.7 (1.0–14.0)	0.051

**Abbreviation:** CI = confidence interval.

* Adjusted odds ratios estimated by logistic regression model compare odds of “yes” among participants in any training with no training. Models controlled for agency/governance type, staff member size, volunteers, client volume, operating budget, borough, and religious affiliation. P-values <0.05 were considered statistically significant.

^†^ Emergency resources include transportation, radios, emergency food supplies, and other essential emergency supplies.

^§^ Organizations have dedicated funding for use during emergencies.

Among the 185 faith-based organizations that responded to the survey, 57 (31%) participated in at least one training (range = 1–6). After controlling for judicatory operation, religious affiliation, clergy size, congregation size, client volume, budget, and borough, participation in at least one community preparedness program training significantly increased the odds of having plans for continuity of operations (AOR = 2.5, p = 0.037), emergency management (AOR = 7.2, p<0.001), volunteer management (AOR = 4.5, p = 0.004), and emergency communications (AOR = 2.8, p = 0.011) in faith-based organizations (Table 2). Community preparedness training also significantly increased the odds of having an emergency notification system (AOR = 3.4, p = 0.001); inventoried emergency resources (AOR = 4.5, p<0.001); the ability to communicate with persons at risk (AOR = 2.1, p = 0.043) before, during, and after an emergency; and dedicated emergency funds (AOR = 3.8, p = 0.013).

**TABLE 2:** Participation in community preparedness program training among faith-based organizations (N = 185) — New York City, 2018

Preparedness component in place	No. (%) responding “yes”		Adjusted odds ratio* (95% CI)	P-value
	Participated in any training (n = 57)	Did not participate in any training (n = 128)		
Continuity of operations plan	20 (35)	14 (11)	2.5 (1.06–6.07)	0.037
Emergency management plan	18 (32)	8 (6)	7.2 (2.8–18.3)	<0.001
Plan for using volunteers	15 (26)	8 (6)	4.5 (1.6–12.4)	0.004
Emergency communications plan	23 (40)	20 (16)	2.8 (1.3–6.1)	0.011
Emergency notifications system	31 (54)	27 (21)	3.4 (1.6–7.3)	0.001
At-risk population communication	32 (56)	45 (35)	2.1 (1.0–4.1)	0.043
Inventory of emergency resources^†^	22 (39)	14 (11)	4.5 (2.0–10.0)	<0.001
Dedicated emergency funds^§^	10 (18)	7 (5)	3.8 (1.3–10.8)	0.013

**Abbreviation:** CI = confidence interval.

* Adjusted odds ratios estimated by logistic regression model compare odds of “yes” among participants in any training with no training. Models controlled for judicatory operation, religious affiliation, clergy size, congregation size, client volume, budget, and borough. P-values <0.05 were considered statistically significant.

^†^ Emergency resources include transportation, radios, emergency food supplies, and other essential emergency supplies.

^§^ Organizations have dedicated funding for use during emergencies.

## Discussion

In many instances, community-based organizations are the first to provide critical recovery services to their communities after a disaster (*1*). It is important that as trusted neighborhood partners, these organizations maintain sufficient levels of preparedness such as those examined in this survey (*2*–*4*). These findings suggest that focused preparedness training might enhance organizational capacity for developing a written plan for continuity of operations that identifies essential services and clearly outlines roles and responsibilities needed to maintain essential operations. In addition, the trainings contributed to more organizations planning for emergency and volunteer management in the event of a disaster, and trainings improved organizations’ planning for emergency communications.

Having documented continuity of operations plans can ensure that community-based organizations are able to maintain essential services following a disaster. Plans should include how volunteers, who frequently contribute to a community-based organization’s daily operations, are recruited and integrated during an emergency response. Because many community-based organizations are trusted information hubs for their service catchment areas, delineating strategies that facilitate the communication of timely and accurate information during an emergency could reduce uncertainty and confusion for staff members and their constituents (*2*). In addition, community-based organizations are encouraged to designate resources and allocate funding for specific use during emergencies to continue to provide essential services.

The findings in this report are subject to at least two limitations. First, survey participants were not randomly selected and response rates were low (*6*,*7*). Human services organizations and faith-based organizations were only invited to complete the online surveys by e-mail, with reminder e-mails sent every 2 weeks, and no incentives were provided. Employing multiple invitation methods and incentives might have improved survey response rates (*6*,*7*). Second, results were not adjusted for any potential selection or nonresponse bias. Therefore, results might not be generalizable to human services organizations and faith-based organizations outside NYC. Nonetheless, results indicate that NYC’s community preparedness program training might improve preparedness in both human services and faith-based sectors.

Community-based organizations can serve as bridges between public health systems and communities and between communities and persons within those communities. Organizations’ familiarity with local communities puts them in a position to identify and address specific requirements for responding to public health emergencies based on their knowledge of available resources, the population, and community needs (*1*,*2*,*8*,*9*). Participation in community preparedness training was associated with higher preparedness levels among NYC human services organizations and faith-based organizations. The NYC community preparedness program model might serve as an example for local health departments seeking methods to engage communities and strengthen readiness for an increasing range and intensity of disasters (*3*). Having community-based organizations meet preparedness standards might increase critical support to many socially and economically diverse communities during emergencies and increase the possibility of saving lives and reducing morbidity following a large-scale disaster.

**Summary**

**What is already known about this topic?**

Human services organizations and faith-based organizations have a long history of engagement in public health issues and can contribute to building community disaster preparedness.

**What is added by this report?**

New York City (NYC) organizations that participated in community preparedness program training had significantly increased odds of having plans for continuity of operations, emergency management, volunteer management, and emergency communication than did those that did not participate in training.

**What are the implications for public health practice?**

The NYC community preparedness program can serve as a model for other health departments considering training community-based organizations to support community preparedness for responding to public health disasters.
